# Nanoparticles and Neurotoxicity

**DOI:** 10.3390/ijms12096267

**Published:** 2011-09-23

**Authors:** Tin-Tin Win-Shwe, Hidekazu Fujimaki

**Affiliations:** 1 Center for Environmental Health Sciences, National Institute for Environmental Studies, 16-2 Onogawa, Tsukuba, Ibaraki 305-8506, Japan; 2 Center for Environmental Risk Research, National Institute for Environmental Studies, 16-2 Onogawa, Tsukuba, Ibaraki 305-8506, Japan; E-Mail: fujimaki@nies.go.jp

**Keywords:** nanoparticles, brain, neurotoxicity, neuroinflammation, oxidative stress

## Abstract

Humans are exposed to nanoparticles (NPs; diameter < 100 nm) from ambient air and certain workplaces. There are two main types of NPs; combustion-derived NPs (e.g., particulate matters, diesel exhaust particles, welding fumes) and manufactured or engineered NPs (e.g., titanium dioxide, carbon black, carbon nanotubes, silver, zinc oxide, copper oxide). Recently, there have been increasing reports indicating that inhaled NPs can reach the brain and may be associated with neurodegeneration. It is necessary to evaluate the potential toxic effects of NPs on brain because most of the neurobehavioral disorders may be of environmental origin. This review highlights studies on both combustion-derived NP- and manufactured or engineered NP-induced neuroinflammation, oxidative stress, and gene expression, as well as the possible mechanism of these effects in animal models and in humans.

## 1. Introduction

In this review the term “nanoparticles” (NPs) is used to define particle less than 100 nm in diameter. NPs are generally classified as combustion-derived NPs (e.g., particulate matters (PM), diesel exhaust particles, welding fumes) and manufactured or engineered NPs (e.g., titanium dioxide, carbon black, carbon nanotubes, silver, zinc oxide, copper oxide). Although these two types of NPs might be the same size, there are obvious differences between them. Combustion-derived NPs are polydispersed, chemically complex nature, soluble or poorly soluble form and toxicity may be due to physicochemical characteristics on their surface [1]. In contrast, manufactured or engineered NPs are monodispersed, precise chemical nature, solid mainly spherical and may be tube, fiber or wire form [1]. Regarding combustion-derived NPs, several epidemiological studies have demonstrated that exposure to elevated levels of PM in urban air is associated with adverse health effects in humans, including an increase in morbidity and mortality related to pulmonary and cardiovascular diseases in susceptible populations [2–5]. Recently, the toxicity of diesel engine-derived NPs has come to be recognized as an emerging social problem. Currently, many manufactured or engineered commercial NPs have been used in electronics, cosmetics, medicines, fabrics, and computer displays [1,6–8]. However, research on the potential health risks of exposure to NPs lags behind the rapid development of nanotechnology. Intratracheal instillation of commercial carbon black (CB) NPs causes pulmonary inflammation and these NPs are translocated to mediastinal lymph nodes and have been shown to size-specifically upregulate expression of chemokine mRNAs in the lungs and lymph nodes [9]. A rat study has shown that ^13^C NPs may be taken up directly into the brain (the olfactory bulb) from the olfactory epithelium via the olfactory nerves [10], and other studies have shown that the nanosized component of PM can reach the brain and may be associated with neurodegenerative diseases [11,12]. Some NPs are difficult to eliminate by physiological clearance systems and may accumulate within the brain over long periods and trigger toxic effects [13].

NPs of various chemicals have been found to be more toxic and inflammogenic than micro-sized particles of the same chemicals when delivered at an equal mass dose [14,15]. Although *in vivo* and *in vitro* studies have shown that combustion-derived NPs are neurotoxic, it has been difficult to evaluate hazard assessment of manufactured NPs, because these NPs readily become aggregations [16]. In this review, we highlight research on both of combustion-derived NP- and manufactured or engineered NP-induced neuroinflammation and neurotoxicity and the possible mechanisms of the effect of NPs in animal models and in human.

## 2. Nanoparticles and Translocation to the Brain

Recently, there has been increasing incidence of neurodegenerative diseases such as Alzheimer’s disease [17], Parkinson’s disease, Huntington’s disease [18], and primary brain tumors [19]. The exact etiology of these diseases is unknown, but environmental pollutants, including NPs, may be an important risk factor. NPs may enter the human body by various routes—including inhalation, injection, dermal penetration, and ingestion—and may then be distributed by means of systemic circulation to various tissues [20,21], perhaps including the brain.

There has been some argument about whether or not NPs can cross the blood–brain barrier (BBB), which separates blood from cerebrospinal fluid and is made of endothelial cells connected by tight junctions that limit the entry of many substances into the brain [22]. However, direct disruption of neuronal cell membranes by NPs would allow their entry into the brain [23,24]. For example, intravenous, intraperitoneal, or intracerebral administration of Ag, Cu, or Al NPs (50–60 nm) reportedly disrupts the BBB, as indicated by staining with albumin-bound Evans blue [23].

Several studies have suggested that the olfactory nerve pathway should be considered as a portal of entry to the central nervous system in humans who are environmentally or occupationally exposed to airborne NPs [10,25,26]. In a landmark study in 1970, De Lorenzo used ultrafine particle to demonstrate that, in squirrel monkeys, intranasally instilled Ag-coated colloidal Au particles (50 nm) translocate anterogradely in the axons of the olfactory nerves to the olfactory bulbs [25]. It has been also shown that Mn, Cd, Ni, and Co nanomaterials that come into contact with the olfactory epithelium can be transported to the brain via the olfactory neurons [27–31]. Oberdörster and colleagues demonstrated that inhalation of ultrafine elemental ^13^C particles (36 nm) by rats for 6 h in a whole-body exposure chamber leads to a significant and persistent increase in the accumulation of ^13^C NPs in the olfactory bulb on day 1 and that the NP concentration continues to increase up to day 7 [10]. The same study also showed that concentrations of ^13^C NPs are significantly increased in the cerebrum and cerebellum but that the increase is inconsistent; that is, it is significant only on one additional day of the post-exposure period (day 1).

In another study, 30-nm manganese oxide NPs were found in different parts of rat brain after intranasal instillation, and the investigators suggested that the NPs had moved to the brain by the olfactory neuronal pathway [31]. It has also been shown that mRNAs of proinflammatory cytokines and chemokines are induced in the olfactory bulb, but not in the hippocampus, of mice intranasally instilled with 14-nm CB particles [32]; these particles might translocate to the olfactory bulb via olfactory neurons and activate major immune cell microglia, which in turn up-regulates the expression of proinflammatory cytokine and chemokine mRNAs. Taken together, it is suggested that the NPs might be involved in inflammation in the brain.

## 3. Nanoparticles and Oxidative Stress

### Combustion-derived NPs

PM that includes NPs can trigger free radical activity on the surface of the particles [33–35]. Free radicals or oxidative stress may damage lipids, nucleic acids, and proteins at the site of particle deposition and at translocation sites, and the brain is particularly vulnerable to oxidative stress because of its high energy demand, low level of antioxidants, and high cellular content of lipids and proteins [36]. Block and colleagues reported that NPs from diesel engine exhaust can damage dopaminergic neurons in primary central nervous system cultures via significantly high levels of free radicals produced by microglial activation [11]. ROS and oxidative stress have been experimentally implicated in the pathogenesis of neurodegenerative disorders. Further studies of oxidative stress associated with selective gene expression analyses and immunological biomarkers would improve our understanding of the mechanisms of neuroinflammation and neurodegeneration associated with NPs.

### Manufactured or engineered NPs

Oxidative stress induced by functionality on the surface of NPs plays a critical role in the common mechanism of their toxicity [10,37] and triggers inflammation in the organs in which NPs are deposited. [38]. NPs such as C60 fullerenes, single-walled carbon nanotubes, quantum dots, and ultrafine particles produce reactive oxygen species (ROS) especially when exposed to light (particularly UV light) or transition metals [39–48]. For example, Ag-25 NPs generate ROS and induce oxidative DNA damage in the brain. [49]. Exposure of mice deficient in apolipoprotein E to concentrated ambient NPs results in enhanced levels of oxidative stress in the brain [36]. ROS are reportedly associated with neurodegenerative disorders such as Parkinson’s disease, Alzheimer’s disease, and Huntington’s disease [18]. Moreover, a recent study indicated that maternal exposure to titanium oxide (TiO_2_) NPs alters the expression level of genes related to apoptosis and oxidative stress in newborn mouse pups [50].

## 4. Nanoparticles and *in Vivo* Animal Studies

### Combustion-derived NPs

In 2005, it was first reported that the levels of the proinflammatory cytokines interleukin (IL)-1α and tumor necrosis factor (TNF)-α and the transcription factor NF-κB are higher in brain tissues of mice exposed to PM than in brain tissues of control animals [51]. These findings suggest that components of PM may trigger an inflammatory response in nervous tissue and that they may contribute to the pathophysiology of neurodegenerative diseases.

The results of a study conducted at the National Institute for Environmental Studies, Japan, indicated that 1-month exposure to NP-rich diesel exhaust (NRDE; generated from an Hino diesel engine (J08C; Hino Motors Ltd., Hino, Japan)) and/or weekly injection of LTA may separately induce neurotoxic effects by modulating extracellular glutamate levels and expression of N-methyl-D-aspartate (NMDA) receptor subunits and related kinases and transcription factors in the mouse olfactory bulb [52]. Insoluble or poorly soluble NPs can enter the brain by nasal route via neuronal transsynaptic transport [10,31], and uptake through the BBB from systemic circulation [23,53]. NPs from diesel exhaust might be translocated to the olfactory bulb, where they stimulate neurotransmitter release from neurons such as periglomerular cells, tuft cells, mitral cells, and granule cells into the extracellular fluid. Increased extracellular glutamate levels may be due to enhanced synthesis, reduced reuptake, or blockade of various types of glutamate receptors on the postsynaptic membrane. Impairment of glutamate uptake by the glutamate transporter is a possible candidate in the NRDE-induced increase in extracellular glutamate levels, which in turn enhances expression of the NMDA receptor subunit mRNA in the olfactory bulb [52].

In another study, expression in mouse hippocampus of genes related to spatial learning ability and memory function was examined after exposure of the animals to NRDE for 1 month with or without a bacterial cell wall component [54]. The relative mRNA levels of the NMDA receptor subunits and proinflammatory cytokines were higher in the hippocampus in the NRDE/LTA (+) group, and poor learning performance was observed. However, NRDE exposure alone did not affect gene expressions and learning performance. Excitatory amino acids, especially glutamate, may induce neuronal damage mediated by abnormal activation of specific NMDA receptor subunits in the hippocampus, leading to impairment of spatial learning, and upregulation of NMDA receptor subunit expression may be due to a compensatory response to reduction in the number of functional receptors.

In a recent study under physiologically relevant conditions, female BALB/c mice were exposed to clean air, a moderate dose (35 μg/m^3^) of NRDE (M-NRDE), a high dose (122 μg/m^3^) of NRDE (H-NRDE), or filtered diesel exhaust (F-DE) for 3 months in the absence of LTA [55]. Mice exposed to H-NRDE took longer to reach a hidden platform and showed higher levels of expression of the mRNAs of the NMDA receptor subunit NR2A, the proinflammatory cytokine CCL3, and brain-derived neurotrophic factor (BDNF) in the hippocampus relative to expression in the control group. Interestingly, the level of BDNF mRNA expression, but not that of NGF mRNA expression, was upregulated in the groups exposed to H-NRDE and F-DE, however, no significant difference in BDNF mRNA was observed between the these two groups. The fact that the concentration of gases such as CO, SO_2_, NO*_x_*, NO_2_, NO, and CO_2_ in the H-NRDE and F-DE chambers were approximately the same indicates that the effect may have been due to the gaseous constituents of the diesel exhaust rather than to NPs [55]. The H-NRDE dose was slightly higher than the environmental quality standard for suspended particulate matter in Japan (100 μg/m^3^). These findings show that subchronic, high-dose NRDE exposure affects expression of genes related to hippocampal-dependent spatial learning and memory function in female mice. However, whether the H-NRDE concentration used presents a danger to human cognitive function is not known.

Developmental nanotoxicological studies have shown that gestational or neonatal inhalation exposure to diesel exhaust during a critical period of brain development affects sexual differentiation by modulating the expression of estrogen receptors α and β in the mouse cerebrum [56]. Furthermore, intrauterine exposure to low levels of diesel exhaust reduces spontaneous locomotor activity and alters the levels of monoamine neurotransmitters such as dopamine and noradrenaline and their metabolites in different brain regions of 4- to 5-week-old mice [57].

### Manufactured or engineered NPs

The mRNAs of IL-1β and TNF-α, chemokines (monocyte chemoattractant protein-1/CCL2 and macrophage inflammatory protein-1α/CCL3), and momokine-induced interferon-gamma/CXC chemokine ligand (CXCL9) are induced in the olfactory bulb, but not in the hippocampus, of mice intranasally instilled with 14-nm CB [32]. Alteration of neurotransmitter levels and proinflammatory cytokines in the mouse olfactory bulb after intranasal instillation of 14-nm CB NPs with or without the bacterial cell wall component lipoteichoic acid (LTA) derived from *Staphylococcus aureus* was also reported [58]. Extracellular glutamate and glycine levels and IL-1β mRNAs were upregulated in the olfactory bulbs of CB-instilled mice, and LTA enhanced these effects [58]. Moreover, low levels of ultrafine PM exposure elicited inflammatory responses mediated by MAP kinase pathways, and high levels of exposure lead to cell death [59]. A recent *in vivo* study in ICR mice showed that aluminium oxide (alumina) NPs induced apoptosis via increased caspase-3 gene, and impaired spatial learning behavior and suggested that mitochondrial impairment plays a key role in neurotoxicity of nano-alumina [60]. It was demonstrated that nano-sized TiO2 (20–100 nm) was detected in cerebral cortex, hippocampus and olfactory bulb of 6-week-old male mice by using field emission-type scanning electron microscopy (FE-SEM) after prenatal exposure [61]. Regardless of dosage and route of administration, once NPs enter the blood stream of a pregnant mother mouse, they could move to the offspring brain through under-developed blood-brain-barrier [62,63].

*In vivo* experiments are necessary to assess sensitivity of organ system to NPs, as well as pharmacokinetic factors, the time course of brain development, and cell-to-cell interactions that cannot be modeled *in vitro*.

## 5. Nanoparticles and *in Vitro* Studies

### Combustion-derived NPs

*In vitro* study has shown that microglia are activated by DEP to produce extracellular superoxide through NADPH oxidase, which is selectively toxic to dopmine neurons [11]. It has been also shown that DEP impair the blood–brain barrier and cause capillaries to release TNFα *in vitro*, contributing to inflammation [64]. Moreover, co-exposure of DEP (5 μg/mL) with lipopolysaccharide (2.5 ng/mL) in primary neuron–glia cultures synergistically increased nitric oxide production, TNF-α release, and dopamine neurotoxicity [65].

### Manufactured or engineered NPs

Several *in vitro* studies have indicated the potential toxicity of NPs to various types of neuronal and glial cells. PC12 neuronal cell lines are commonly used for neurobiological and neurochemical assessment of NP-induced neurotoxicity. Exposure of PC12 cells to 40-nm manganese oxide NPs dose-dependently depleted dopamine and its metabolites, dihydroxyphenylacetic acid and homovanillic acid and those depletion were associated with significantly increased production of ROS [66]. Furthermore, exposure to 0.15- to 15-nm anionic magnetic NPs triggered dose-dependent diminished viability of PC12 cells using methyl-thiazol-tetrazolium (MTT) method [67]. In addition, Wang and colleagues (2009) showed that dopamine system-related gene expression is altered in PC12 cells exposed to Mn (40 nm), Ag (15 nm), or Cu (90 nm) NPs [68]. These investigators found that the Cu NPs induce dopamine depletion in PC12 cells and that the Mn NPs induce a similar effect; Wang and colleagues suggested that the dopaminergic neurotoxicity induced by the Mn and Cu NPs may share some mechanisms associated with neurodegeneration. A recent *in vitro* study using a dopaminergic PC12 cell line indicated that SiO2-NPs decreased cell viability, triggered oxidative stress, disturbed cell cycle, induced apoptosis and the p53 mediated signaling pathway [69].

BV2 is an immortalized mouse microglial cell line commonly used to assess cellular toxicity for pharmacological agents, PM, and environmental chemicals [11,70]. Exposure of BV2 cells to Degussa P25, a commercially available TiO_2_ nanomaterial, results in immediate and prolonged release of ROS and upregulation of inflammatory, apoptotic, and cell cycling pathways, as well as downregulation of energy metabolism [71]. Apart from PC12 and BV2 cell lines in nanotoxicology, a recent study indicated that exposure to ZnO NPs in mouse neural stem cells induced cell apoptosis [72].

*In vitro* studies have some limitations; for example, they do not permit assessment of developmental and functional events in a single individual. Nevertheless, *in vitro* nanotoxicological studies are necessary for clarification of the mechanism of action of NPs without the influence of nutrition, endocrine status, and other variables. Therefore, a combination of both *in vivo* and *in vitro* studies would improve our ability to assess the risks of various NPs.

## 6. Nanoparticles and Human Studies

### Combustion-derived NPs

Human data on the potential health hazards of NP exposure under real-world conditions is limited. Human studies carried out in Mexico City indicate that exposure to severe air pollution including PM is associated with brain inflammation via increased production of cyclooxygenase-2 (an inflammation mediator) and accumulation of a 42-amino-acid form of β-amyloid (an Alzheimer’s disease marker) [73].

Some reports indicating a statistically significant association between ambient ultrafine particle concentrations and increased risk of cardiopulmonary morbidity and mortality has been published [74,75]; however, interpretation of this association remains problematic [76]. One major problem is that separating NPs from other toxicologically relevant PM2.5 and PM10 fractions and gaseous components (nitrogen oxides, carbon monoxide, ozone) of the complex air mixture is difficult, and exposure measurement errors are introduced by the use of central site monitoring data in place of personal exposure data [74].

Diesel exhaust particles are a major constituent of ambient PM, and most particles emitted directly from diesel exhaust are smaller than 1 μm in diameter [77,78]. In a clinical study on the effects of diesel exhaust on humans, healthy young male volunteers were exposed for 1 h to whole diesel exhaust from a Volvo Diesel engine (Volvo TD45, 4.5 L, 4 cylinders, 680 rpm) during exposure and for 1 h after exposure, brain electrical activity was monitored by means of quantitative electroencephalography [79]. A significant increase in median power frequency in the frontal cortex within 30 min exposure was observed in these volunteers. These changes in brain activity may have been associated with NPs that either penetrated the brain or affected neurophysiologic signaling [79]. Toxicologically relevant physicochemical properties of NPs from diesel exhaust may differ from the properties of other NPs [76], and it is suggested that different NPs have a specific neurotoxic and neurobehavioral effects.

### Manufactured or engineered NPs

A few clinical studies have shown that laboratory-generated NPs, such as elemental carbon and zinc oxide NPs, adversely affect pulmonary health [80,81]. The first evidence of NP-related disease in humans was found in seven Chinese workers in a print factory where a polyacrylic ester paste containing NPs was used; the workers suffered from an unusual and progressive lung disease; two of the workers died of the disease [82].

Further studies are necessary to assess the human health risks of ambient and engineered NPs and to aid in the establishment of regulations regarding exposure to NPs.

## 7. Conclusions

**Figure 1:**
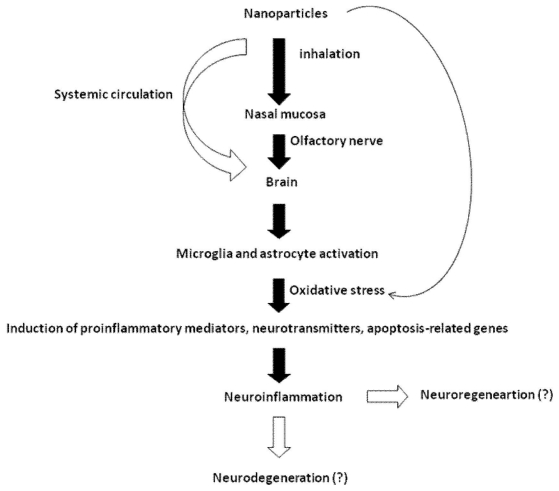
Potential pathways of nanoparticles-induced neurotoxicity.

On the basis of our findings and those of other investigators, we have summarized various pathways for NP-induced neurotoxicity in Figure 1. Although numerous *in vivo* and *in vitro* studies have provided evidence of the toxic effects of various types of NPs, our understanding of the potential health and safety issues regarding NPs lags behind the rapid commercialization of nanomaterials. The benefits of nanomaterials must be weighed against their potential toxic effects. One major problem is lacking information on the possible adverse health effects caused by exposure to different nanomaterials [83]. Therefore, understanding of the neurotoxic effects of manufactured or engineered NPs would help in the development of safety guidelines by authorities to promote nanotechnology for applications without hazard.

Figure 1. Potential pathways of nanoparticle-induced neurotoxicity. NPs deposited in the nasal mouse may enter the brain via olfactory bulb. Another portal of entry of NPs to brain is from systemic circulation. In the brain, NPs may induce inflammation, apoptosis and oxidative stress by releasing various mediators from microglia and astrocyte. Depends on production of toxic (e.g., NO, excitatory neurotranmsitters) or anti-toxic mediators (e.g., anti-inflammatory cytokines, neurotrophins), it may lead to neurodegeneration or neuroregeneration.
